# The heat-shock response co-inducer arimoclomol protects against retinal degeneration in rhodopsin retinitis pigmentosa

**DOI:** 10.1038/cddis.2014.214

**Published:** 2014-05-22

**Authors:** D A Parfitt, M Aguila, C H McCulley, D Bevilacqua, H F Mendes, D Athanasiou, S S Novoselov, N Kanuga, P M Munro, P J Coffey, B Kalmar, L Greensmith, M E Cheetham

**Affiliations:** 1 Ocular Biology and Therapeutics, UCL Institute of Ophthalmology, London, UK; 2 Sobell Department of Motor Neuroscience and Movement Disorders, UCL Institute of Neurology, London, UK; 3 MRC Centre for Neuromuscular Diseases, UCL Institute of Neurology, Queen Square, London, UK

**Keywords:** retinal degeneration, neurodegeneration, cell stress, molecular chaperone, protein aggregation, rhodopsin

## Abstract

Retinitis pigmentosa (RP) is a group of inherited diseases that cause blindness due to the progressive death of rod and cone photoreceptors in the retina. There are currently no effective treatments for RP. Inherited mutations in rhodopsin, the light-sensing protein of rod photoreceptor cells, are the most common cause of autosomal-dominant RP. The majority of mutations in rhodopsin, including the common P23H substitution, lead to protein misfolding, which is a feature in many neurodegenerative disorders. Previous studies have shown that upregulating molecular chaperone expression can delay disease progression in models of neurodegeneration. Here, we have explored the potential of the heat-shock protein co-inducer arimoclomol to ameliorate rhodopsin RP. In a cell model of P23H rod opsin RP, arimoclomol reduced P23H rod opsin aggregation and improved viability of mutant rhodopsin-expressing cells. In P23H rhodopsin transgenic rat models, pharmacological potentiation of the stress response with arimoclomol improved electroretinogram responses and prolonged photoreceptor survival, as assessed by measuring outer nuclear layer thickness in the retina. Furthermore, treated animal retinae showed improved photoreceptor outer segment structure and reduced rhodopsin aggregation compared with vehicle-treated controls. The heat-shock response (HSR) was activated in P23H retinae, and this was enhanced with arimoclomol treatment. Furthermore, the unfolded protein response (UPR), which is induced in P23H transgenic rats, was also enhanced in the retinae of arimoclomol-treated animals, suggesting that arimoclomol can potentiate the UPR as well as the HSR. These data suggest that pharmacological enhancement of cellular stress responses may be a potential treatment for rhodopsin RP and that arimoclomol could benefit diseases where ER stress is a factor.

Retinitis pigmentosa (RP) is a group of heterogeneous disorders characterised by progressive rod photoreceptor loss followed by cone cell death in the retina.^1^ There are currently no effective therapies for RP. *Rhodopsin* was the first RP gene identified,^2^ and mutations in rhodopsin are the most common cause of autosomal-dominant RP (RetNet). Rhodopsin is formed of rod opsin protein and the chromophore 11-*cis*-retinal. The most common point mutation in North America is the proline to histidine change at residue 23, hereafter referred to as P23H. Rod opsin mutations have been classified according to their cellular and biochemical characteristics.^3^ Class II rod opsin mutations are the most common and include P23H, which is misfolded and retained in the endoplasmic reticulum (ER), occasionally forming visible intracellular inclusions of aggregated protein.^4, 5^ There is also evidence that the P23H mutation has a dominant-negative effect on wild-type rhodopsin^4, 6, 7^ and can affect outer segment (OS) disc formation and stability.^8, 9^

Within the cell, a network of dedicated protein-folding mechanisms function to assist proteins attain their correct conformation and perform quality control on misfolded or damaged proteins, thereby establishing protein homeostasis or proteostasis.^10^ These mechanisms include the heat-shock response (HSR) and unfolded protein response (UPR). In the HSR, following cell stress molecular chaperones, such as Hsp70 and Hsp90, dissociate from their transcription factor heat-shock factor 1 (HSF1) and bind misfolded proteins. HSF1 is posttranslationally modified and trimerises before trafficking to the nucleus to induce molecular chaperone expression.^11, 12^ There are three distinct branches of the UPR that respond to ER stress: IRE1*α*, PERK, and ATF6*α*.^13, 14^ BiP (HSPA5), the resident Hsp70 orthologue in the ER, regulates all three branches. IRE1*α* (inositol requiring enzyme-1) activation results in the correct splicing of XBP1, which can act as a transcription factor to upregulate ER chaperones and ER-associated degradation (ERAD) components. ATF6 (activating transcription factor 6) traffics to the Golgi, where it is cleaved, and the N-terminal fragment enters the nucleus, where it upregulates molecular chaperones and quality-control components. PERK (double-stranded RNA-activated protein kinase (PKR)-like ER kinase) autophosphorylation results in eIF2*α* phosphorylation, which leads to the inhibition of protein synthesis. However, selective transcription of certain genes still occurs; the transcription factor ATF4 is one of these, which leads to upregulation of the pro-apoptotic protein CHOP and phosphatase GADD34. In cells with high protein turnover, these networks are particularly important to preserve proteostasis. Rod photoreceptors produce large amounts of rhodopsin, and mutant rhodopsin can induce the UPR,^15^ highlighting the role of these networks in adapting to protein misfolding stress in photoreceptors.^16^

Much recent work has focused on the role of these networks to alleviate protein misfolding diseases, especially the targeted upregulation of these mechanisms to reduce protein aggregation and restore proteostasis. Hsp90 inhibition is a potent method for inducing the HSR. Hsp90 inhibitors, such as geldanamycin and its analogues, bind the ATP-binding pocket of Hsp90 and block its ATPase cycle, thus releasing HSF1, which can activate heat-shock protein expression.^17, 18^ Inhibition of Hsp90 can reduce P23H rhodopsin inclusion formation, insolubility and reduce P23H rod opsin-associated cell death.^19^ Recently, Hsp90 inhibition was shown not only to reduce P23H rhodopsin-mediated photoreceptor cell death *in vivo* but can also potentially affect normal visual function, suggesting that other mechanisms to induce the stress response might be desirable.^20^

The hydroxylamine derivative bimoclomol is a co-inducer of the HSR, which can potentiate a pre-existing HSR and enhance the expression of inducible molecular chaperones (e.g., Hsp70) when combined with a cellular stress event such as heat shock, ischemia, or pancreatitis.^21, 22^ Interestingly, bimoclomol treatment also resulted in a functional improvement in a rat model of diabetic retinopathy, with increased b-wave electroretinogram (ERG) amplitude compared with vehicle-treated rats.^23^ Arimoclomol (also known as BRX-345) is an analogue of bimoclomol and a citrate formulation of a previously used maleate formulation analogue (BRX-220), which has a longer half-life and better uptake than bimoclomol. In a rat sciatic nerve crush model, treatment with BRX-220 increased motor neuron survival.^24^ Arimoclomol also improved motor neuron survival in both the SOD^G93A^ mouse model of amyotrophic lateral sclerosis (ALS)^25, 26^ and a mouse model of spinal bulbar muscular atrophy, a CAG-repeat disorder.^27^ Bimoclomol and its analogues BRX-220 and arimoclomol have been shown to prolong and increase the activation of HSF1,^25, 28^ which leads to increased upregulation of Hsp70 and Hsp90, *in vitro*^21^ and *in vivo*.^25, 26^

In the present study, we investigated the effect of arimoclomol treatment on *in vitro* and *in vivo* models of P23H rhodopsin RP. Arimoclomol treatment reduced inclusion incidence and cell death in a cell model. Furthermore, arimoclomol treatment in P23H rats preserved retinal function and photoreceptor survival. We show that these effects are related to the potentiation of both the HSR and UPR.

## Results

### Arimoclomol reduces P23H aggregation and improves cell viability in SK-N-SH cells

**Figure 1:**
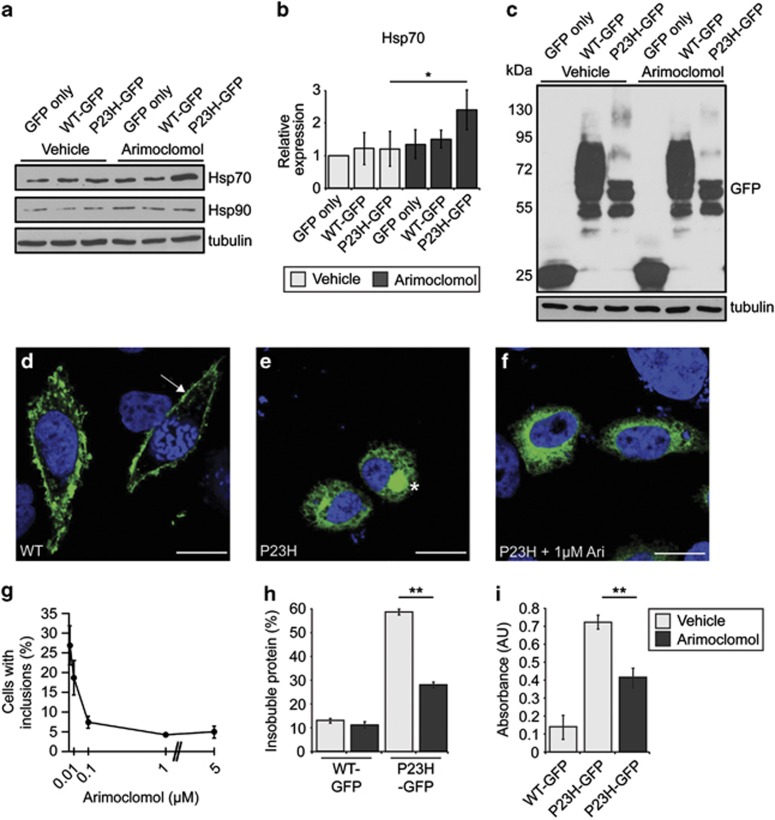
Arimoclomol induces the HSR and reduces aggregation of P23H-GFP in cells. (**a**) Representative western blots of SK-N-SH cell lysates transfected with either GFP alone or WT- or P23H-GFP, and treated with vehicle (water) or 1 *μ*M arimoclomol for 24 h, for Hsp70, Hsp90, or tubulin, as indicated. (**b**) Quantification of expression levels of Hsp70 in transfected SK-N-SH cells, normalised to tubulin. Relative expression to vehicle-treated cells transfected with GFP only was calculated by densitometric analysis. Values are mean±2 × S.E.M. Statistical significance was determined using analysis of variance, *P<0.05. (**c**) Western blots for GFP for cells transfected with WT- or P23H-GFP and treated as indicated. Molecular weight markers (in kDa) are indicated to the left of blots. Tubulin was used as a loading control. (**d**–**f**) Representative images of WT-GFP, P23H-GFP and P23H-GFP treated with 1 μM arimoclomol localisation in cells. Arrow in panel (**d**) indicates the presence of WT-GFP at the plasma membrane, whereas asterisk in panel (**e**) indicates intracellular inclusion formed by P23H-GFP. Scale bar 10 *μ*m. (**g**) P23H-GFP-expressing cells were treated with increasing concentrations of arimoclomol, and inclusion incidence was assessed. Values are mean±2 × S.E.M. (**h**) WT- and P23H-GFP cells treated with 1 *μ*M arimoclomol were examined for the amount of insoluble sedimentable protein present using a rod opsin fractionation assay. Values are mean±2 × S.E.M., and statistical significance was determined using the Student's *t*-test, **P<0.01. (**i**) Cells were subjected to LDH release assay to assess cell death. Values are mean±2 × S.E.M., and statistical significance was determined using the Student's *t*-test, following removal of GFP background, **P<0.01

Pharmacological chaperones, kosmotropes and molecular chaperone inducers can affect rod opsin folding and aggregation in cell models.^19, 20^ Arimoclomol is an HSR potentiating compound; therefore, we initially investigated the effect of arimoclomol on HSR activation in SK-N-SH cells expressing wild-type or P23H rod opsin tagged with GFP (WT-GFP and P23H-GFP, respectively). A significant increase in Hsp70 level was observed in P23H-GFP-expressing cells treated with arimoclomol compared with vehicle-treated P23H-GFP cells (Figures 1a and b). Furthermore, there appeared to be a small decrease in the amount of higher molecular weight species of P23H-GFP rod opsin in treated cells compared with untreated cells (Figure 1c), potentially corresponding to higher-order complexes of rod opsin. As previously reported,^4^ WT-GFP trafficked to the plasma membrane (Figure 1d), whereas P23H-GFP was retained in the ER, and occasionally formed intracellular inclusions (Figure 1e). Arimoclomol treatment led to a dose-dependent decrease in the incidence of cells with inclusions, from 27% in vehicle treated P23H-GFP expressing cells to <5% in cells treated with 1 or 5 *μ*M arimoclomol (Figures 1f and g). Similarly, P23H-GFP cells treated with 1 μM arimoclomol had reduced amounts of sedimentable, insoluble rod opsin (Figure 1h). Arimoclomol did not affect WT-GFP expression. Furthermore, P23H-GFP cells treated with arimoclomol had reduced cell death compared with vehicle-treated P23H-GFP cells, as measured by a lactate dehydrogenase (LDH) assay (Figure 1i).

### Arimoclomol treatment improves visual function in P23H rats

**Figure 2:**
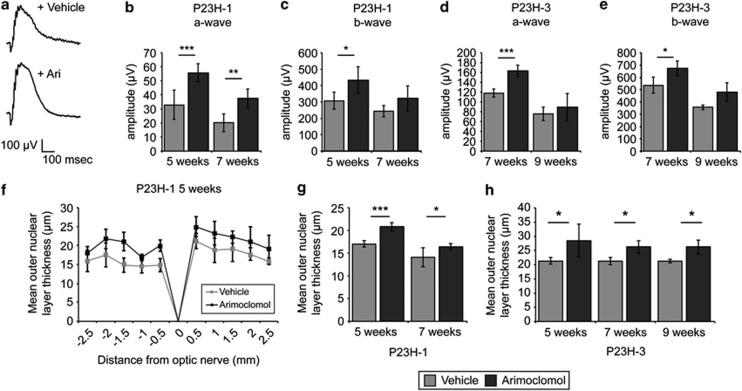
Arimoclomol improves ERG response and photoreceptor survival in P23H rats. (**a**) Representative scotopic ERG traces of P23H-1 rats at P35 after treatment with vehicle (water) or 10 mg/kg arimoclomol for 2 weeks. (**b** and **c**) P23H-1 and (**d** and **e**) P23H-3 rats were treated with either vehicle or arimoclomol (*n*=5 for each condition) and were assessed for retinal function at 5, 7, or 9 weeks, as indicated. Values represent mean scotopic ERG response for a- and b-waves. Intensity=1 log10 c.d.s./m^2^±2 × S.E.M. Statistical significance was determined using the Student's *t*-test, *P<0.05, **P<0.01, ***P<0.001. (**f**–**h**) P23H-1 and P23H-3 rats were treated with either vehicle (water) or 10 mg/kg arimoclomol (*n*=5 for each condition) and were assessed for alterations in retinal histology at 5, 7, or 9 weeks, as indicated. (**f**) Spider plot of ONL thickness in P23H-1 rats at 5 weeks after vehicle or arimoclomol treatment. (**g** and **h**) The mean ONL thickness across the whole retina was analysed by averaging the measurements used in spider plots. Values are mean±2 × S.E.M. Statistical significance was determined using the Student's *t*-test, *P<0.05, **P<0.01, ***P<0.001

Given the positive effect of arimoclomol on mutant rod opsin observed in this cell model, we investigated arimoclomol treatment in an *in vivo* model, using two lines of the P23H rat.^29^ Line 1 P23H (P23H-1) rats undergo rapid photoreceptor degeneration, while line 3 P23H (P23H-3) have a markedly slower degeneration.^30, 31^ All animals were treated daily with 10 mg/kg arimoclomol by intraperitoneal injection, a dose that has been shown to be effective in SOD1^G93A^ mice.^25^ P23H-1 rats were treated from P21 (3 weeks of age) to either P35 (5 weeks of age) or P49 (7 weeks of age), while P23H-3 rats were treated from P21 to either P49 or P63 (9 weeks of age). All animals were tested for visual function improvements by scotopic ERG measurements (Figure 2). Both a- and b-wave response amplitudes were improved in P23H-1 and P23H-3 rats following arimoclomol treatment (Figures 2b–e), compared with vehicle-treated animals.

### Arimoclomol treatment protects against P23H-mediated photoreceptor degeneration

**Figure 3:**
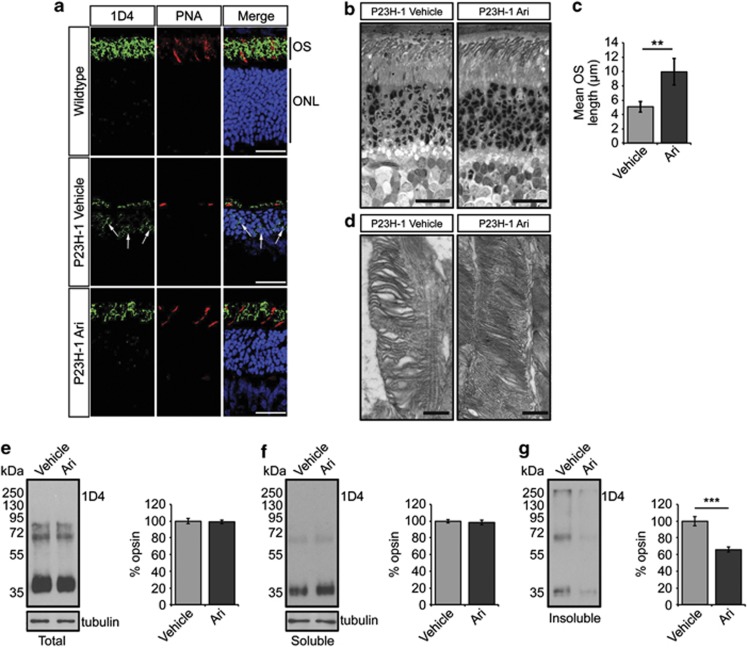
Arimoclomol treatment improves OS structure, rhodopsin localisation and solubility in P23H-1 rats. (**a**) Representative images of the ONL in either 5-week-old wild-type, P23H-1 rats treated with vehicle (water) or 10 mg/kg arimoclomol for 2 weeks. Cryosections were stained with DAPI (blue), anti-rhodopsin antibody 1D4 (green), and cone marker peanut agglutinin (PNA; red). Scale bar 20 *μ*m. (**b**) Representative images of semi-thin resin sections of 5-week-old P23H-1 retina stained with toluidine blue. Scale bar 10 *μ*m. (**c**) Quantification of OS length in vehicle- or arimoclomol-treated P23H-1 rats. Values represent mean±2 × S.E.M., *n*=3 per treatment. Statistical significance was determined using the Student's *t*-test, **P<0.01. (**d**) Representative TEM images of vehicle- and arimoclomol-treated P23H-1 retina showing the structure of the rod OS. Scale bar 500 nm. (**e**–**g**). Representative western blots and densitometric analysis of (**e**) total, (**f**) soluble, and (**g**) insoluble rod opsin levels in vehicle- or arimoclomol-treated P23H-1 rats at 5 weeks. Molecular weight markers (in kDa) are indicated to the left of blots. Tubulin was used as a loading control. Values represent mean±2 × S.E.M., *n*=3 per treatment. Statistical significance was determined using the Student's *t*-test ***P<0.001

After ERG examinations, eyes were excised and examined for changes in retinal architecture by histological analysis. We measured outer nuclear layer (ONL) thickness as a measure of photoreceptor survival. In P23H-1 rats, ONL thickness was preserved across the entire retina in treated animals compared with vehicle-treated animals (Figure 2f), and mean ONL thickness was significantly maintained at all time points in both strains (Figures 2g and h). Rhodopsin expression was investigated in retinae of both wild-type Sprague Dawley (SD) rats and treated and vehicle-treated P23H-1 animals at P35. As expected, SD rat retinae showed rhodopsin expression in the OS (Figure 3a); however, in P23H retinae there was a marked reduction of OS rhodopsin staining and increased rhodopsin expression in the ONL, which suggests that P23H rhodopsin is not trafficked correctly and might affect the traffic of wild-type rhodopsin (Figure 3a, arrows). In arimoclomol-treated rats, there appeared to be an increase in OS length as assessed by rhodopsin staining and a decrease in ONL rhodopsin staining (Figure 3a). Further examination of arimoclomol-treated and vehicle-treated eyes in semi-thin resin-embedded sections revealed a significant increase in OS length in arimoclomol-treated animals, from approximately 5 *μ*m to 10 *μ*m (Figures 3b and c). Transmission electron microscopy (TEM) was used to analyse the rod structure in more detail. P23H-1 rats had a disorganised rod OS structure with loosely packed, misorientated discs and more vesicular structures, whereas in arimoclomol-treated animals the general disorganisation was reduced (Figure 3d). Interestingly, the amount of total and soluble rhodopsin was found to be similar in both vehicle- and arimoclomol-treated eyes when normalised to tubulin (Figures 3e and f), but fractionation analysis of the retina revealed that there was a significant reduction in insoluble rhodopsin in arimoclomol-treated animals (Figure 3g).

### Arimoclomol potentiates pre-existing stress responses in P23H-1 rats

**Figure 4:**
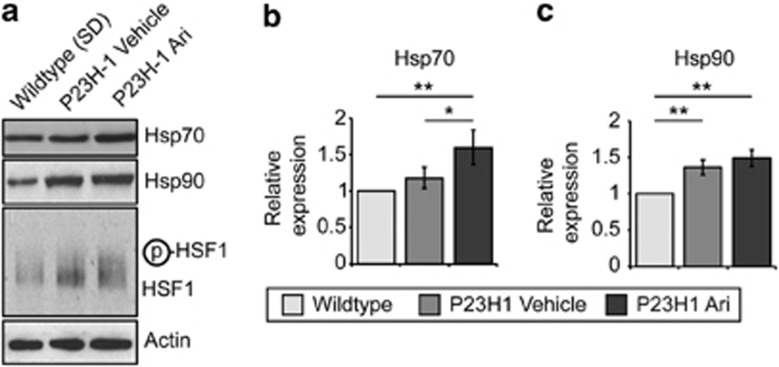
Arimoclomol treatment co-induces the HSR in P23H-1 rats. (**a**) Representative western blots of retinal lysates from P23H-1 rats at 5 weeks old treated with vehicle (water) or 10 mg/kg arimoclomol for 2 weeks. Antibodies against the HSR markers Hsp70, Hsp90, and HSF1 are shown, as indicated. Actin was used as a loading control. (**b** and **c**) Quantification of the expression levels of (**b**) Hsp70 and (**c**) Hsp90 levels in wild-type or vehicle- or arimoclomol-treated P23H-1 rats, relative to actin. Relative expression to wild-type rats was calculated by densitometric analysis. Values are mean±2 × S.E.M., *n*=5 per treatment. Statistical significance was determined by using analysis of variance, *P<0.05, **P<0.01

**Figure 5:**
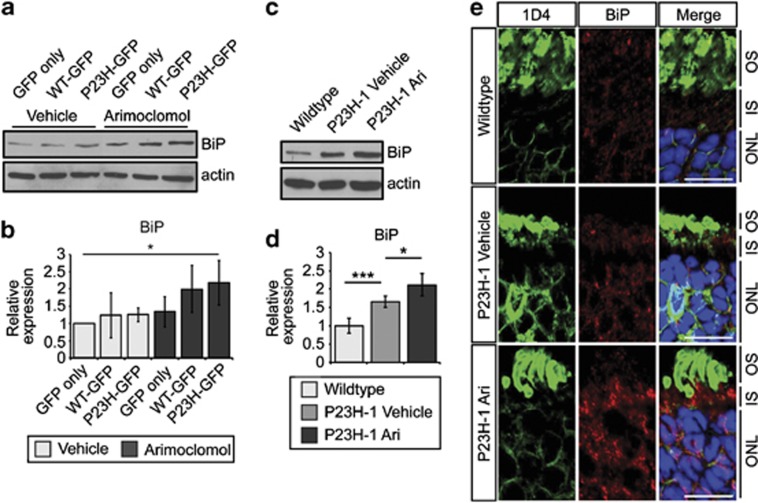
Arimoclomol treatment potentiates upregulation of BiP in P23H-1 rats. (**a**) Representative western blots of BiP expression in lysates from SK-N-SH cells transfected with GFP only, WT-GFP, or P23H-GFP after 24 h of treatment with vehicle of 1 μM arimoclomol. Actin was used as a loading control. (**b**) Quantification of the BiP expression levels in SK-N-SH cells in panel (**a**), relative to actin. Relative expression to wild-type rats was calculated by densitometric analysis. Values are mean±2 × S.E.M. Statistical significance was determined by using analysis of variance (ANOVA), *P<0.05. (**c**) Representative western blots of BiP expression in retinal lysates from wild-type or vehicle- or arimoclomol-treated P23H-1 rats at P35. (**d**) Quantification of the BiP expression levels in wild-type or vehicle- or arimoclomol-treated P23H-1 rats, relative to actin. Relative expression to wild-type rats was calculated by densitometric analysis. Values are mean±2 × S.E.M., *n*=5 per treatment. Statistical significance was determined by using ANOVA, *P<0.05, ***P<0.001. (**e**) Representative identical intensity images of BiP immunohistochemistry in wild-type or vehicle- or arimoclomol-treated P23H-1 rats at P35. Cryosections were stained with DAPI (blue), anti-rhodopsin antibody 1D4 (green), and anti-BiP (red). The different layers of the retina are indicated to the right of the image: OS; IS=inner segment; and ONL. Scale bar 10 *μ*m

**Figure 6:**
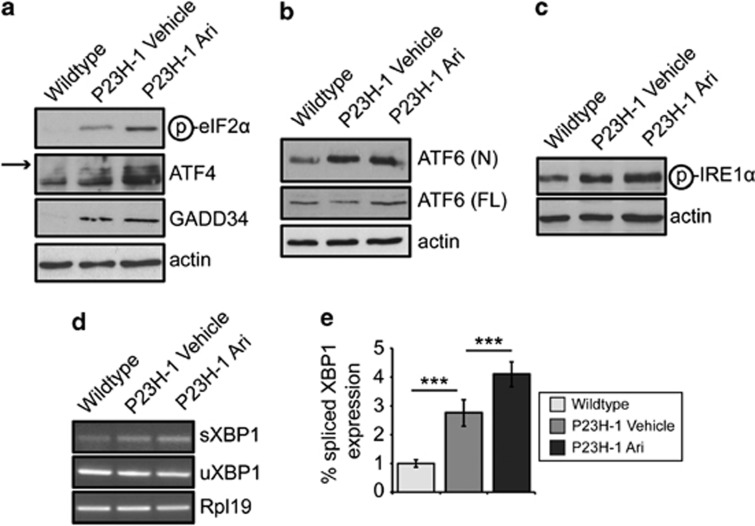
Arimoclomol treatment potentiates upregulation of the UPR in P23H-1 rats. (**a**–**c**) Representative western blots of retinal lysates from wild-type or P23H-1 rats at P35 for antibodies against the three branches of the UPR: (**a**) the PERK branch (phosphorylated eIF2*α*, ATF4 (arrow indicates specific ATF4 band) and GADD34); (**b**) the ATF6 branch (cleaved or nuclear (N) ATF6 and full-length (FL) ATF6); and (**c**) the IRE1α branch (phosphorylated IRE1*α*). Actin was used as a loading control throughout. (**d**) RT-PCR using primers targeting spliced XBP1 and unspliced XBP1 in cDNA from P23H-1 retinae at 5 weeks. Rpl19 was used as a normalisation control. (**e**) Quantification of RT-PCR using primers targeting spliced XBP1 and unspliced XBP1 in cDNA from P23H-1 rats at P35. The ratio of spliced XBP1 to unspliced XBP1 was normalised to Rpl19 levels and normalised to wild-type XBP1 ratio. Statistical significance was determined by using analysis of variance, *P<0.05, ***P<0.001

Arimoclomol potentiated the HSR in the presence of P23H-GFP in cells (Figure 1); therefore, we investigated whether arimoclomol enhanced the HSR in P23H-1 rats. P23H-1 rat retinae at P35 had slightly elevated levels of Hsp70 and Hsp90, and an increase in posttranslationally modified HSF1 with a slower mobility compared with SD control animals, suggesting a pre-existing induction of the HSR (Figure 4a). Treatment with arimoclomol further increased Hsp70 and Hsp90 expression levels and increased mobility shifted HSF1 (Figures 4a and b). As the P23H mutation causes rod opsin misfolding and retention in the ER and has been reported to induce the UPR,^15, 32^ we examined P23H rod opsin models for changes in the ER-resident molecular chaperone BiP, which is a marker of the UPR. In SK-N-SH cells expressing WT- or P23H-GFP, BiP levels were slightly elevated in untreated P23H-GFP cells, although this increase was not significant (Figures 5a and b). Arimoclomol treatment led to a significant increase in BiP levels in P23H-GFP cells compared with untreated cells expressing GFP only. In P23H-1 rat retinae BiP levels were increased at P35, compared with SD controls, and a further increase was observed following arimoclomol treatment (Figures 5c and d). Furthermore, this increase was confirmed and localised by immunohistological analyses of wild-type and P23H retinae. BiP levels were markedly increased in the inner segment and ONL of the photoreceptors (Figure 5e). Interestingly, the levels of BiP changed most dramatically in the ONL of P23H-1 and arimoclomol-treated P23H-1 retinae, whereas there were higher levels of constant expression in the bipolar cells (in the inner nuclear layer) and retinal ganglion cells (Supplementary Figure S1) in all animals. The UPR has three distinct branches (IRE1*α*, PERK, and ATF6*α*), each with different downstream effects.^13^ Therefore we also examined several UPR markers from each branch (Figure 6 and Supplementary Figure S2). Western blotting analysis showed that levels of phosphorylated eIF2*α*, ATF4, GADD34, and cleaved ATF6*α* (ATF6-N) were all increased in P23H-1 retinae compared with SD retinae, supporting induction of the UPR, as previously reported.^15^ In the arimoclomol-treated P23H-1 retinae, these UPR markers were also significantly increased compared with SD. In addition, the increase in phosphorylated IRE1*α* reached statistical significance (Supplementary Figure S2). Most of the UPR markers were elevated in P23H-1 retinae treated with arimoclomol compared with vehicle but did not reach statistical significance; however, there was a significant increase in phosphorylated eIF2*α* (Figures 6a–c and Supplementary Figure S2). XBP1 splicing was examined via a RT-PCR assay using primers specifically targeting the spliced intron (Figure 6d). P23H-1 retinae showed increased XBP1 splicing compared with wild type, and a further increase after arimoclomol treatment was observed (Figure 6e). These data are consistent with arimoclomol treatment enhancing the activation of all three branches of the UPR in the presence of P23H mutant rod opsin.

## Discussion

Arimoclomol and related hydroximic acid derivatives (HADs) have been shown to be protective in several models of disease. Here, we investigated the therapeutic potential of arimoclomol for P23H rhodopsin-mediated retinal degeneration. In SK-N-SH cells, arimoclomol reduced P23H aggregation but did not lead to improved trafficking of P23H rod opsin. However, this was sufficient to reduce P23H rod opsin-mediated cell death. This correlation between reduced rod opsin aggregation and improved cell viability was also observed for kosmotropes, Hsp90 inhibitors,^19^ and BiP overexpression.^32, 33^ Furthermore, the rescue of P23H misfolding by molecular chaperones or pharmacological chaperones has revealed that the protein is intrinsically unstable.^34, 35^ Collectively, these data suggest that enhancing P23H rod opsin solubility and improved protein quality control may be more important than improving protein folding to counteract the toxic effects of mutant rod opsin.

It has been shown that rhodopsin expression is reduced in P23H-1 transgenic rats from at least postnatal day 30 (P30) and rod degeneration starts as early as P16.^30, 36^ Similarly, OS length is decreased by 40% in P40 P23H-1 animals,^30^ and retinal sensitivity correlates with OS length in human RP patients,^37^ suggesting that changes in the OS might precede and predict photoreceptor cell loss. P23H rhodopsin cannot sustain normal OS biogenesis in the absence of wild-type rhodopsin, and heterozygous P23H knock-in mice have disrupted disc organisation in their OS.^8, 9, 38^ The reasons for this ultrastructural disruption are not clear at present but might correspond to insufficient rhodopsin for proper disc formation, a toxic gain of function of the mutant protein that disrupts the disc structure or a combination of both these processes. P23H rhodopsin has a dominant-negative effect on wild-type rhodopsin,^4, 6, 7^ and therefore its presence can lead to lower levels of both wild-type and mutant rhodopsin through ER retention and degradation. Furthermore, P23H-GFP expression in *Xenopus* rods disrupted discs and destabilised the OS structure.^39^ We observed an increase in photoreceptor survival, OS length, and improved OS organisation in arimoclomol-treated rats, suggesting that arimoclomol treatment can counteract the dominant effects of P23H rhodopsin. Importantly, this was not due to an increase in the amount of soluble rhodopsin, suggesting that the OS disruption seen in this model is at least partly due to a toxic gain of function of the P23H protein, which was counteracted by arimoclomol treatment. These data suggest that preservation of the retinal structure and photoreceptor survival lead to improved visual responses in arimoclomol-treated animals.

Arimoclomol enhanced the activation of HSF1 and expression of Hsp70 in the presence of P23H rod opsin in both cells and in the retina. This suggests that misfolded P23H rod opsin can initiate the HSR, in addition to the published induction of the UPR.^15^ This activation of the HSR was potentiated by arimoclomol. Interestingly, the induction of the HSR through HSF1 activation can protect against P23H rod opsin aggregation and promote cell viability in cells and in the retina,^19, 20^ suggesting a potential role in dealing with misfolded rhodopsin by the HSR. However, the downstream targets of the HSR that protect against P23H rod opsin RP are not known at present. It is possible that there are specific molecular chaperones that target mutant rod opsin, or it is possible that it is the co-ordinated action of several induced chaperones that help reduce aggregation and/or promote survival.

Interestingly, we observed that arimoclomol treatment not only potentiated the HSR but also enhanced the UPR; therefore the upregulation of these dual stress responses may co-operate to protect photoreceptor cells. There is the potential for crosstalk between the HSR and the UPR. For example, in yeast, mild heat shock or constitutively active Hsf1 can rescue growth defects associated with lack of functional Ire1, by increasing the levels of BiP.^40^ The HSR is also induced by tunicamycin treatment in cells lacking Ire1.^40^ In addition, heat stress can activate the UPR in mammalian cells via eIF2*α* phosphorylation and XBP1 splicing.^41^ Nevertheless, the data presented here are the first demonstration that arimoclomol can potentiate the UPR when in the presence of a misfolded protein in the ER and suggest that pharmacological manipulation of the HSR and UPR can be integrated to protect cells from stress.

The HSR and UPR feed downstream to the proteasome and/or autophagy for destruction of misfolded cytosolic or ER proteins. ER proteins are destroyed by ERAD, which has been implicated in P23H rod opsin degradation. EDEM1 recognises and binds P23H rhodopsin and targets it for ERAD.^34^ The clearance of P23H rhodopsin is also regulated by the ERAD effector protein VCP.^42^ Interestingly, arimoclomol treatment resulted in less P23H rod opsin aggregation in cells and the photoreceptors of transgenic rats without an increase in overall rhodopsin levels, suggesting an enhancement of aggregation-prone rod opsin degradation.

All three branches of the UPR are activated by the dissociation of BiP from their lumenal domains in response to detection of unfolded or misfolded protein. BiP has also been implicated in P23H rhodopsin RP. BiP mRNA and protein are increased in rat models of rhodopsin RP.^43, 44^ Furthermore, BiP associates with rod opsin in the ER, and overexpression of BiP improved P23H rod opsin solubility.^33^ Indeed, overexpression of BiP via subretinal AAV delivery in P23H-3 rats increased BiP levels in the retina and preserved retinal function and ONL thickness.^32^ BiP levels were increased following arimoclomol treatment, suggesting that some of the protective effect might be mediated by increased BiP function.

The UPR is a protective response to ER stress, but prolonged activation can promote apoptotic cell death.^13^ Therefore, the induction of the UPR, and in particular the PERK branch, has been implicated in both the protection against misfolded proteins and also in cell death. For example, in a SOD^G85R^ model of ALS, the expression of mutant GADD34, which lacks the phosphatase domain, improved disease progression, probably owing to increased eIF2*α* phosphorylation repressing translation.^45^ Similarly, PERK haploinsufficiency (i.e., mice heterozygous for a PERK null mutation) decreased survival in SOD^G85R^ mice, as eIF2*α* phosphorylation was reduced.^46^ Conversely, increased phosphorylated eIF2*α* in prion diseased mice has been linked with increased neuronal cell death, and overexpression of GADD34 significantly reduced neuron loss.^47^ Similarly, PERK inhibition in prion mice reduced eIF2*α* phosphorylation and slowed disease progression,^48^ suggesting that PERK activation and translational blockade by eIF2*α* phosphorylation contributes to neuronal cell death. Another downstream target of PERK is the pro-apoptotic protein CHOP, which has been implicated in initiating cell death following ER stress;^14^ however, ablation of CHOP did not protect photoreceptors against P23H or T17M mutant rhodopsin.^49, 50^ BiP expression is upregulated by ATF4 activity after increased eIF2*α* phosphorylation,^51^ hence, in the short term, enhanced PERK activity may also be protective. We observed increased eIF2*α* phosphorylation after arimoclomol treatment, which might contribute to the elevated levels of BiP in combination with IRE1 activation and XBP1 splicing. Furthermore, we did not see an increase in GADD34 levels after arimoclomol treatment, suggesting that decreased dephosphorylation may contribute to increased phosphorylated eIF2*α*. Therefore, PERK activation and eIF2*α* phosphorylation are not necessarily markers that predict photoreceptor cell death in rhodopsin RP, and our data suggest that enhancement of the UPR does not lead to photoreceptor cell death but instead might be protective.

HADs, like arimoclomol, are well documented as heat shock co-inducers. For example, bimoclomol has been shown to upregulate the HSR in models of ischemia,^21^ and BGP-15 co-induces the HSR in induced-neuropathy models.^52^ BGP-15 has also been shown to improve prognosis in a mouse model of Duchenne muscular dystrophy^53^ and is currently in clinical trials for diabetes.^54^ However, the precise mode of action of these compounds is still unclear. Bimoclomol has been shown to increase the activation of HSF1, probably by prolonging phosphorylation,^28^ and BGP-15 reduced acetylation of HSF1, also prolonging its activation.^55^ HADs can modify membrane fluidity in response to heat shock, thus activating downstream signalling targets of the HSR,^56^ and research has focussed on the role of HADs at the plasma membrane, in particular lipid rafts, detergent-resistant membrane regions that have a role in sensing cellular stress.^57^ The ability of arimoclomol to potentiate UPR induction by P23H-GFP also suggests that it is active at the ER membrane.

This study has shown that potentiation of both the HSR and UPR via arimoclomol treatment can alleviate the damage caused by the rhodopsin P23H mutation in P23H rats. This approach might be more effective than stimulating the HSR or UPR alone. Furthermore, because the effect of arimoclomol is dependent on the preexisting stress provided by the misfolded rod opsin, there might be fewer side effects related to the alteration of the proteostasis machinery other than other compounds, such as Hsp90 inhibitors.^19, 20^ The ability of arimoclomol to enhance multiple branches of the cell stress machinery will also potentially expand the range of diseases for this drug and other HADs.

## Materials and Methods

### Reagents and antibodies

Arimoclomol was synthesised by BioBlocks (Budapest, Hungary). Rod opsin-GFP plasmids were as previously described.^4^ Lipofectamine and Plus reagent were purchased from Invitrogen (Paisley, UK). Protease inhibitor cocktail (PIC), phosphatase inhibitor cocktail (PhIC), and 4',6-diamidino-2-phenylindole dihydrochloride (DAPI) were from Sigma (Poole, UK).

1D4 mouse monoclonal antibody (mAb) against rod opsin (1.33 mg/ml) was a gift from Professor Robert Molday (University of British Columbia, Vancouver, BC, Canada) and used at a dilution of 1 : 1500 in all applications. Hsp70 mouse mAb (1 :1 000) and Hsp90 rat mAb (1 : 1000) were from Stressgen (Ann Arbor, MI, USA). GFP mouse mAb (1 : 1000) was from Roche (Burgess Hill, UK). HSF1 rat mAb (1 : 1000) was from Abcam (Cambridge, UK). *β*-Tubulin mouse mAb (1 : 5000), BiP rabbit polyclonal antibody (pAb; 1 : 3000), and phosphorylated eIF2*α* (pSer51) rabbit pAb (1 : 1000) were from Sigma. ATF4 rabbit pAb (1 : 1000) and ATF6 rabbit pAb (1 : 1000) were from Santa Cruz (Santa Cruz, CA, USA). Phosphorylated IRE1*α* (pSer724) rabbit pAb (1 : 1000) was from Novus Biologicals (Cambridge, UK). Actin (clone C4) mouse mAb (1 : 5000) was from Millipore (Watford, UK). GADD34 rabbit pAb (1 : 1000) was from Proteintech (Manchester, UK). Rhodomine-conjugated peanut agglutinin was from VectorLabs (Peterborough, UK). Goat anti-mouse Alexa Fluor 488 and goat anti-rabbit Alex Fluor 594 secondary antibodies conjugated IgGs (1 : 1000) were from Invitrogen. Goat anti-mouse, anti-rat, and anti-rabbit secondary antibodies conjugated to horseradish peroxidase were from Pierce (Cramlington, UK).

### Animals and arimoclomol treatment

Wild-type SD rats were purchased from Harlan (Blackthorn, UK). P23H-1 and P23H-3 line rats were kindly provided by Professor Matt LaVail (UCSF, San Francisco, CA, USA). Animals were housed in 12 : 12 light/dark cycle with food and water available *ad libitum*. Arimoclomol was dissolved in water and delivered to animals via intraperitoneal (i.p.) injection. Animals were treated with arimoclomol at 10 mg/kg daily as indicated. All procedures were conducted according to the UK Home Office regulations under the Animals (Scientific Procedures) Act 1986, and with local UCL Institute of Ophthalmology (London, UK) ethics committee approval.

### Electroretinography

Scoptopic ERG was performed as previously described.^20, 58^ Briefly, animals were dark-adapted overnight before being anaesthetised via ketamine/xylazine i.p. injection. ERG was performed using platinum loop electrodes on the cornea, and flash stimuli were presented via LED stimulator. All procedures were carried out under red-light conditions with the animal on a 37 °C heated pad.

### Cell transfection and fluorescence

SK-N-SH cells were cultured and transfected as previously described.^19^ Unless indicated, cells were treated with 1 μM arimoclomol for 24 h and added to the cell culture media directly after transfection. Immunofluorescence fixing and DAPI staining was performed as previously described.^20^ LDH activity assay was performed as previously described.^19^

### Immunohistochemistry

For OS measurement studies, eyes from rats were fixed in 3% v/v glutaraldehyde and 1% w/v paraformaldehyde buffered to pH 7.4 with 0.08 M sodium cacodylate-HCl. Fixed eyes were dissected to isolate the anterior segment and lens. The posterior segments were then rinsed in 0.1 M sodium cacodylate buffer at pH 7.4, osmicated with 1% aqueous osmium tetroxide, and dehydrated through ascending alcohols (50–90%) and left overnight at room temperature on a rotator in a 1 : 1 mixture of propylene oxide/absolute alcohol. Posterior segments were embedded and cured overnight at 60 °C. Semithin and ultrathin sections were cut using a Leica (Milton Keynes, UK) Ultracut S microtome fitted with the appropriate type of diamond knife. For light microscopy, semithin sections were stained with 1% toluidine blue and 1% borax in 50% ethanol. Ultrathin sections were contrasted for 5 min with 1% uranyl acetate in 50% ethanol followed by Reynold's lead citrate, before viewing and photographing with a JEOL (Welwyn Garden City, UK) 1010 TEM operating at 80 kV.

For fluorescence studies, eyes were removed from rats at specified time points and fixed overnight in 4% paraformaldehyde at 4 °C. Postfixation eyes were cryoprotected by incubation in 30% sucrose in PBS. Eyes were then frozen and cyrosectioned as described previously.^20^ Cryosectioned eyes were incubated in blocking buffer (3% bovine serum albumin (BSA) and 10% normal goat serum in PBS) for 1 h at room temperature before incubation with primary antibodies as indicated. DAPI staining was used to visualise the nuclei.

### Imaging and analysis

Cell and retina images were obtained using the Carl Zeiss (Cambridge, UK) LSM700 laser-scanning confocal microscope. Images for OS length analysis were taken on a Carl Zeiss LSM510 confocal microscope. Images were exported from the Zen 2009 (Carl Zeiss) software and prepared using Adobe (San Jose, CA, USA) Photoshop and Illustrator CS4. Cell morphology studies were scored as previously described.^19^ Images for ONL thickness measurements were taken on a Nikon (Kingston-upon-Thames, UK) Eclipse 80i. ONL thickness measurements were made on digital images of stained cryosections, every 500 μm from the optic nerve outwards for both the inferior and superior hemisphere. Results from five animals at each time point were averaged and represented as a spider plot. All measurements were performed in ImageJ (http://rsbweb.nih.gov/ij/), and subsequent analysis was performed in Microsoft Excel (Redmond, WA, USA).

### Western blotting and sedimentation assays

Western blotting was performed as previously described.^20^ Briefly, SK-N-SH cells were lysed in ice-cold 1% DM buffer with 2% PIC. Frozen rat retinae were lysed in ice-cold RIPA buffer with 2% PIC and 2% PhIC. The opsin solubility assay in transfected SK-N-SH cells was performed as previously described.^19^ The opsin fractionation assay using transgenic rat retinae was performed as previously described.^20^ All samples were separated by SDS-PAGE and analysed by western blotting. Primary antibodies were diluted in 5% BSA in Tris-buffered saline with 0.05% Tween20 (TBST), and incubation was carried out overnight at 4 °C using the antibodies listed in Materials and Methods. After scanning developed films, densitometry analysis was completed using ImageJ. The average pixel density was measured for each band.

### RNA extraction and RT-PCR

Frozen retina were subjected to RNA extraction using the RNeasy Mini Kit (Qiagen, Crawley, UK), and cDNA synthesis was performed via reverse transcription (RT) using the SuperScript III First-Strand Synthesis (Invitrogen) system for RT. Specific amplification of spliced XBP1 was achieved using the following primers: *XBP1sF* (from Hirota *et al*:^59^ 5′-GGTCTGCTGAGTCCGCAGCAGG-3′); and *XBP1sR* (from Samali *et al*:^60^ 5′-GGGTCCAACTTGTCCAGAATGC-3′). Rpl19 was used as an internal reference.^15^ Primers were: *Rpl19F*: 5′-TACCCTTCCTCTTCCCTATGCC-3′ and *Rpl19R*: 5′-TGGACCCCAATGAAACCAAC-3′. GoTaq Green (Promega, Southampton, UK) was used for amplification by PCR with standard cycling conditions.

### Statistical analysis

For the group analysis of western blotting and RT-PCR quantification, one-way analysis of variance and *post-hoc* Tukey's test was used. For the analysis of all other measurements of paired groups (ERG, ONL thickness, and OS length measurements), Student's *t*-test was used. All statistical analysis was performed in either the SPSS (version 22, IBM, Armont, NY, USA) or Microsoft Excel.

## Abbreviations

ER: endoplasmic reticulum
ERG: electroretinogram
HAD: hydroximic acid derivative
HSF1: heat-shock factor 1
HSR: heat-shock response
LDH: lactate dehydrogenase
ONL: outer nuclear layer
OS: outer segment
RP: retinitis pigmentosa
SD: sprague dawley
UPR: unfolded protein response
