# Supplementary material for: The heat-shock response co-inducer arimoclomol protects against retinal degeneration in rhodopsin retinitis pigmentosa
Source: Cell Death Dis. 2014 May 22;5(5):e1236–. doi: 10.1038/cddis.2014.214 (PMC4047904; doi:10.1038/cddis.2014.214)
Supplement: Supplementary Material [file cddis2014214x1.pdf]

Supplementary material

The heat shock response co-inducer arimoclomol protects against retinal degeneration in rhodopsin retinitis pigmentosa

David A. Parfitt, Monica Aguila, Caroline H. McCulley, Dalila Bevilacqua, Hugo F. Mendes, Dimitra Athanasiou, Sergey S. Novoselov, Naheed Kanuga, Peter M. Munro, Peter J. Coffey, Bernadett Kalmar, Linda Greensmith and Michael E. Cheetham

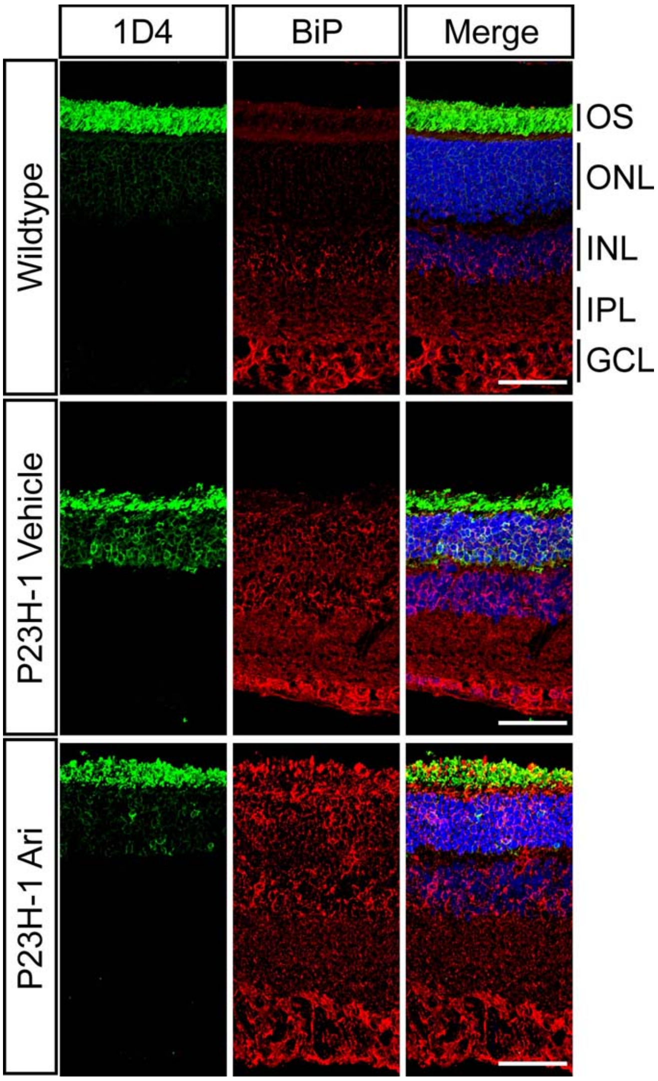

Supplementary Figure 1. Representative images of BiP immunohistochemistry in wildtype or vehicle or arimoclomol treated P23H-1 rats at P35. Cryosections were stained with DAPI (blue), anti-rhodopsin antibody 1D4 (green) and anti-BiP (red). The different layers of the retina are indicated to the right of the image: OS = outer segment; ONL = outer nuclear layer; INL = inner nuclear layer; IPL = inner plexiform layer; GCL= ganglion cell layer. Scale bar 50μm

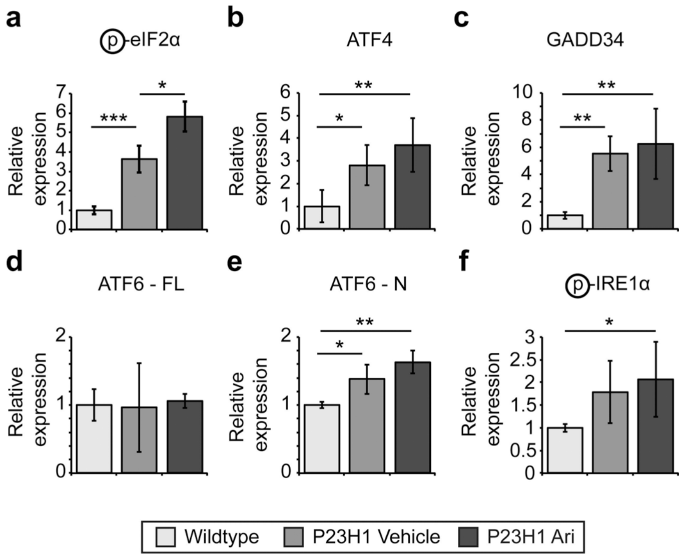

Supplementary Figure 2. Quantification of UPR marker expression levels in wildtype or vehicle or arimoclomol treated P23H-1 rats, relative to actin. Relative expression to wildtype rats was calculated by densitometric analysis. Values are mean ± 2xSEM, *n*=5 per treatment. Statistical significance was determined using ANOVA, * = $p<0.05$, ** = $p<0.01$, *** = $p<0.001$.

.
